# Determinant factors of overweight/obesity among federal ministry civil servants in Addis Ababa, Ethiopia: a call for sector-wise occupational health program

**DOI:** 10.1186/s13104-019-4489-4

**Published:** 2019-07-22

**Authors:** Kassawmar Angaw Bogale, Taye Abuhay Zewale

**Affiliations:** Department of Epidemiology and Bio Statistics, School of Public Health, College of Medicine and Health Sciences, Bahir Dar University, Bahir Dar, Ethiopia

**Keywords:** Overweight and obesity, Determinant factors, Civil servant, Addis Ababa, Ethiopia

## Abstract

**Objective:**

Civil servants are disposing individuals to sedentary lifestyle and, may lead to overweight and obesity. Thus, the purpose of the study was to identify factors associated with overweight and obesity among employees in Ethiopia ministries.

**Result:**

Respondents who were age 45 years and above [AOR = 11.56, 95% CI 3.75–35.56], 35–44 years [AOR = 11.17, 95% CI 3.89–32.06] and 25–34 [AOR = 3.08 95% CI 1.07–8.83] were more likely to be overweight/obesity as compared to those who were in age category of 18–24 years. The study also found that ever alcohol consumption [AOR = 2.27, 95% CI 1.23, 4.16] was associated with increased risk of overweight and obesity as compared to non-consumers. Another risk factor was adult who did not practice ten minutes’ walk per day, more likely to overweight and obesity [AOR = 11.28, 95% CI 5.96–21.36] as compared to the counter parts. Similarly, participants who did not involve physical activity (sport) [AOR = 2.42% 95% CI 1.36–4.30] were 2.42 times more likely to overweight and obesity as compared to those who had physical activity. Sector-wise occupational health program should be developed in work placed to decrease identified risk factors.

## Introduction

World health organizations (WHO) defined overweight/obesity as excessive fat accumulation that may harm health. Body mass index (BMI) is used to classify overweight and obesity in adults commonly. It is a person’s weight in kilograms (kgs) divided by the square of his height in meters (m^2^). Obesity is a BMI greater than or equal to 30; and overweight is a BMI greater than or equal to 25 [1].

Overweight/obesity has been increasing at an alarming rate in both developed and developing countries during the last few decades including in Sub-Saharan Africa [2–4]. Overweight/obesity seems an unusual concern in Ethiopia’s public health, however, the government revealed that being overweight/obesity is emerging fast as a non-communicable public health concern [5, 6]. It leads to serious medical conditions such as hypertension, dyslipidemia, heart attack, stroke, diabetes, gallbladder disease, osteoarthritis, and some cancers [7–9].

Overweight/obesity also increasingly civil servant workers [10, 11]. A study among college of health sciences’ employee in Ghana reported that, the prevalence of overweight and obesity was 43% and 13% respectively [10]. Another study reported that overweight/obesity among bank workers in Ghana was 55.6% [11].

Analysis from Ethiopia Demographic and Health Survey (EDHS) data found that the prevalence of overweight/obesity of adult women in Addis Ababa was 19.9% [12]. Another study in Addis Ababa, Ethiopia showed that the prevalence of overweight/obesity increased by 28% [13].

Civil servants are disposing individuals to the sedentary lifestyle, and some of these jobs are characterized by sitting for a long period of time. Therefore, civil servants become susceptible to developing overweight/obesity. Thus, the purpose of the study was to identify factors associated with overweight and obesity among employees in Ethiopia ministries. The evidence of this study can be used as evidence for program planners, policymakers, researchers, and organizations who are working on the prevention of non-communicable diseases caused by overweight and obesity.

## Main text

### Methods and materials

#### Study design and setting

An institution based cross-sectional study was conducted to identify factors associated with overweight/obesity among ministry civil servants in Addis Ababa which is the capital city of Ethiopia. According to the 2007 population census, the city has a total population of 3,384,569 inhabitants [14]. The city is the seat of the Ethiopian federal government and all ministries. There are twenty-one ministries in Ethiopia and there were 15,808 civil servants in these ministries [15].

#### Study population and sampling method

The study population was all employees who were working in ministries. Pregnant women during the study period were excluded.

The sample size was determined using Epi-Info version 7 software with the assumption of 95% confidence interval, 5% margin of error, design effect 2 and considering 19.9% prevalence of overweight [16] and adding 10% non-response rate which provides a total sample size of 531. Four out of twenty-one ministries namely: Ministry of Urban Construction, Culture and Tourism, Education, and Civil Service were randomly selected. Study participants in four ministries were selected by systematic random sampling method after proportionally allocated to the four ministries.

#### Data collection

Questionnaires were self-completed by respondents adapted from the WHO STEP tool. The data was collected on socio-demographic characteristics (sex, age, educational and marital status, and behavioral factors like alcohol consumption, cigarette smoking, and physical activity) variables were collected [14].

#### Anthropometric measures

Respondents’ body weight and height, measurements were taken. An inelastic measure tape was used to measure participant’s height by standing subjects on bare feet. For weight measurements, participants were standing on an electronic weighing scale with barefoot and light cloths. Then BMI was calculated as BMI = Weight (kg)/[Height (m)^2^]. According to the WHO classification of BMI, Less than 18.5 consider underweight, 18.5–24.9 normal weight, 25–29.9 overweight and ≥ 30 obese [17]. Two BSC nurses were recruited for data collection and took 2 days of intensive training on the objectives of the study, survey procedures and the actual taking of anthropometric measurements.

#### Data analysis

Data were entered into EPI-INFO version 7 software and analyzed by SPSS version 23. Associated factors were identified using binary logistic regression model. Bi-variable analysis was conducted to screen the possible determinants of overweight and obesity and variables with p-values of ≤ 0.2 were included in the multivariable model. Finally, in multi-variable binary logistic regression analysis, factors associated with overweight/obesity were identified using their Adjusted Odds Ratio (AOR) with the corresponding 95% CI.

#### Ethical consideration

The study protocol was reviewed, and ethical clearance was obtained from the Institutional Review Board of the University of Gondar. Official letters were obtained from the administrative body of each ministry. After the purpose of the study has been informed to the study participants, informed written consent was obtained. Confidentiality was maintained by excluding their names in the questionnaire. Those study participants who were over-weighted and obese were advised during data collection.

### Results

#### Socio-demographic and economic characteristics

In this study, a total of 531 participants were recruited of them, 525 participated with the respondent rate of 98.87%. From the respondents 271 (51.8%) were female. Their mean age was 38.36 (± 10.56) years, of them (169, 31.8%) were aged between 25 and 34. Seventy-one percent of respondents were Orthodox religion (377, 71.0%) and three hundred fourteen respondents were married (314, 59%). Regarding education status, two-thirds of respondents were educated to the level of diploma and above (354, 66.5%). About 40% of respondents were paid from 1000 to 2500 per month (210, 40%) (Table 1).

**Table 1:** Socio-demographic characteristics of respondents among ministries civil servants in Addis Ababa, Ethiopia, 2018 (n = 525)

Variable	Category	Frequency	%
Sex (525)	Male	231	44
	Female	294	56
Age (n = 525)	18–25	123	23
	25–34	162	31
	35–44	139	26
	≥ 45	101	19
Religion (n = 525)	Orthodox	373	71.0
	Muslim	51	9.7
	Protestant	87	16.6
	Others	14	2.7
Marital status (n = 525)	Single	179	34.3
	Married	308	59.0
	Divorced	19	3.6
	Widowed	16	3.1
Educational level (n = 525)	Write and read only	24	4.6
	Primary education (1–8)	53	10.2
	Secondary education (9–12)	97	18.7
	Diploma and above	346	66.5
monthly income (ETB) (n = 525)	< 1000	14.3	18.2
	1000–2250	32.3	41.1
	2251–3300	9.2	11.7
	≥ 3301	22.8	29.0

#### Behavioral characteristics

Around 19% of the respondents were reported that they were cigarette smokers (102, 19.3%); of them, 38.4% were currently cigarette smokers (29, 35.3%). On the other way, nearly half of the respondents were reported their past or present alcohol use (49.3%, 259). Eighty-eight respondents were reported chewing chat (88, 17.4%). From this 13% of participants were daily khat chewers (11, 13%). Regarding the dietary status of the respondents, only 7% of the respondents were consumed fruit 4–7 days per week (31, 7%) and 20% (92, 20.2%) of the participants have reported their vegetable consumption 4–7 times per week. About the oil consumption of respondents, 41.6% were utilizing sesame type of oil (205, 41.6%). Regarding physical activity, nearly 70% of the respondents were not involved in vigorous-intensity activities (334, 69.2%) and nearly fifty percent of respondents were not doing sports (253, 48.2%). Similarly, 68.9% of respondents were using a vehicle to go to and come to the home and workplace (348, 68.9%). Whereas, the majority (73%) of the study participants walked at least 10 min per day (382, 73.0%) (Table 2).

**Table 2:** Behavioural and dietary habit of respondents among ministries civil servants in Addis Ababa, Ethiopia, 2018

Variable	Category	Frequency	(%)
Cigarette smoking habit (n = 525)	Yes	103	20.0
	No	422	80.0
Ever consumed an alcoholic drink (n = 525)	Yes	316	50.2
	No	259	49.0
Alcohol consumption frequency in a week (n = 316)	Yes	266	51.0
	No	19	6
Fruit eating habit per week (n = 446)	1–3 days	415	93.0
	4–7 days	31	7.0
Vegetable consumption frequency per week (n = 457)	1–3 days	365	79.9
	4–7 days	92	20.1
Types of oil or fat consumed (n = 525)	Vegetables	212	40.0
	Butter	50	10.0
	Sesame/nug oil	212	40.0
	Others	51	2.0

#### Associated risk factors of overweight and obesity

In the bi-variable analysis age, educational status, income, cigarettes smoking status, chat chewing status, physical activity (sport), and 10 min walk per day and alcohol consumption were filtered. In the multivariable logistic regression analysis variables that showed statistically significant association with obesity and overweight using p-value < 0.05 were age, income alcohol consumption, 10 min walk per day and physical activity (sport). The prevalence of overweight and obesity was increased with age. Respondents who were age 45 years and above [AOR = 11.56, 95% CI 3.75–35.56], 35–44 years [AOR = 11.17, 95% CI 3.89–32.06] and 25–34 [AOR = 3.08 95% CI 1.07–8.83] were more likely to be overweight/obesity as compared to those who were in age category of 24 and 18 years. The odds of overweight and obesity was higher among adult whose monthly income was between 1000 and 2250 [AOR = 6.69, 95% CI 2.02, 22.20] as compared to monthly income between less than 1000. The study also found that ever alcohol consumption [AOR = 2.27, 95% CI 1.23, 4.16] was associated with an increased risk of overweight/obesity as compared to non-consumers. Another risk factor was adults who did not practice 10 min walk per day, more likely to overweight and obesity [AOR = 11.28, 95% CI 5.96–21.36] as compared to their counterparts. Similarly, participants who did not involve physical activity (sport) [AOR = 2.42% 95% CI 1.36–4.30] were 2.42 times more likely to overweight/obesity as compared to those who had physical activity (Table 3).

**Table 3:** Factors associated with overweight/obesity among ministries civil servants in Addis Ababa, Ethiopia, 2018

Variable	Overweight/obesity		COR (95% CI), value	AOR (95% CI)
	Yes	No		
Age of the participant (years)				
18–24	11 (11%)	93 (89%)	1	1
25–34	36 (24%)	117 (76%)	2.60 (1.256, 5.388)	3.08 (1.07–8.83)
35–44	50 (38%)	80 (62%)	5.28 (2.577, 10.83)	11.17 (3.89–32.06)
≥ 45	44 (47%)	50 (53%)	7.440 (3.53, 15.668)	11.56 (3.75–35.56)
Monthly income of participants				
< 1000	5 (6%)	76 (94%	1	1
1000–2250	63 (34%)	120 (66%)	7.98 (3.07, 20.73)	6.69 (2.02, 22.20)
2251–3300	12 (23%)	40 (77%)	4.56 (1.50, 13.85)	1.14 (0.27, 4.75)
≥ 3301	50 (39%)	79 (61%)	9.62 (3.64,25.42)	3.20 (0.90, 11.38)
Ever cigarette smoking				
Smoker	43 (43%)	57 (57%)	2.37 (1.54, 3.70)	1.40 (0.67–2.91)
Non smoker	101 (24%)	318 (76%)	1	1
Ever alcohol drinking				
Yes	95 (37%)	162 (63%)	2.57 (1.72, 3.84)	2.27 (1.23, 4.16)
No	49 (19%)	215 (81%)	1	1
Ever chat chewing status				
Yes	35 (40%)	53 (60%)	1.96 (1.21, 3.17)	
No	105 (25%)	312 (75%)	1	
Physical (sport) activity of respondents				
Yes+	55 (20%)	217 (80%)	2.21 (1.49, 3.27)	2.42 (1.36–4.30)
No	91 (36%)	162 (64%)	1	1
Walk 10 munities per day of respondents				
Yes	64 (17%)	318 (83%)	6.90 (4.49, 10.60)	11.28 (5.96–21.36)
No	82 (58%)	59 (42%)	1	1

Hosmer and Lemeshow test indicate excellent fit, that was p-value greater than 0.5.

### Discussion

Raised BMI is a major risk factor for non-communicable diseases such as cardiovascular diseases (mainly heart disease and stroke), which were the leading cause of death. The risk for these non-communicable diseases increases, with increases in BMI [18]. This study tried to assess the determinate factors of overweight/obesity among ministry civil servants.

Increased age was identified as a factor for overweight and obesity in this study and many other studies [10, 11, 13, 19]. Respondents who were age 45 years and above years were more likely to be overweight/obesity as compared to those who were in the age category of 24 and 18 years. This could be due to the changes in hormone levels with age and also decreasing physical activity with increasing age.

Findings from this study show that alcohol consumption increases the risk of obesity and overweight 2.27 times comparing those who take alcohol to those who do not take alcohol. This finding is in line with the finding in Ghana and China [10, 11, 20]. The possible explanation could be alcohol can increase the risk of weight gain by the calories it provides and by increasing one’s appetite for food.

Another risk factor was adults who did not practice 10 min walk per day, more likely to overweight and obesity and participants who did not involve physical activity (sport) were 2.42 times more likely to overweight and obesity as compared to those who exercise physical activity which is consistent with studies conducted in Namibia, India and Kenya [21–23]. The reason might be participants spend much time sitting and involved less physical labor leads them to a sedentary lifestyle. This might determine the number of calories stored in the body in the form of fat.

Dissimilar many other study reports [21, 24, 25], this finding did not reveal any association of overweight and obesity with cigarette smoking and dietary habits. This could be explained by the difference in the study population, setting, sample size, socio-economic and cultural difference between the two studies.

### Conclusion

Being older age, physically inactive, unable to walk 10 min per day and consumption of alcohol increase the risk of overweight and obesity significantly. Sector wise integrated occupational health program should be developed in the workplace to decrease identified risk factors of overweight/obesity.

## Limitation

The possible limitations of this study were:

- Biochemical measurements were not carried out due to inadequate technical staff and insufficient funds to carry out the work involved.
- The study was a cross-sectional study and the temporal relationships between determinants factor and overweight/obesity cannot be established.
- This study generalizes for ministry civil servants only.

## Data Availability

We will make avail the data based on the request.

## Abbreviations

AOR: Adjusted Odds Ratio
BMI: body mass index
BSC: Bachelor of Science
CI: confidence interval
EDHS: Ethiopia Demographic and Health Survey
EPI-INFO: epidemiological information
kg: kilogram
m: meter
SPSS: Statistical Package for Social Science
STEPS: STEP wise approach to surveillance
WHO: World Health Organization
